# Hybrid staged approach to subclavian artery aneurysm repair with aberrant dominant left vertebral artery

**DOI:** 10.1093/jscr/rjad405

**Published:** 2023-07-29

**Authors:** David J Clausen, John Kanitra, Scott Bendix

**Affiliations:** Department of Surgery, Ascension St John Hospital, Detroit, MI, USA; Department of Surgery, Ascension St John Hospital, Detroit, MI, USA; Department of Surgery, Ascension St John Hospital, Detroit, MI, USA

## Abstract

Intrathoracic subclavian aneurysms are a rare entity, accounting for only a small percentage of all repaired aneurysms. These are repaired to alleviate symptoms and prevent complications of rupture, thrombosis and distal embolization. Most of these are amenable to thoracic endovascular aneurysm repair (TEVAR), which has resulted in an associated reduction in operative mortality. When there is a proximal involvement of the artery, revascularization is recommended prior to TEVAR. Herein, we present the case of a proximal subclavian aneurysm with an aberrant left vertebral artery that originated off the aortic arch. This was repaired using a two staged approach; carotid-subclavian bypass with vertebral artery-transposition followed by TEVAR.

## INTRODUCTION

Intrathoracic subclavian artery aneurysms (SAAs) are rare and account for only 0.5% of all repaired aneurysms [1]. Intervention is performed to alleviate the symptoms of local compression or to prevent rupture, thrombosis and distal embolization [2]. Conventional repair via median sternotomy or thoracotomy has a mortality rate of 8% and a complication rate of 26% [2]. However, the majority of SAAs are amenable to an endovascular approach for repair [3], resulting in a reduction in perioperative mortality [2]. When the proximal portion of the left subclavian artery (LSA) is involved, LSA revascularization prior to or during thoracic endovascular aneurysm repair (TEVAR) is advised [3]. We present our successful management of a unique left SAA in a patient with an aberrant left vertebral artery with concomitant hypoplastic right vertebral artery. This is being presented with the consent of the patient.

## CASE REPORT

**Figure 1:**
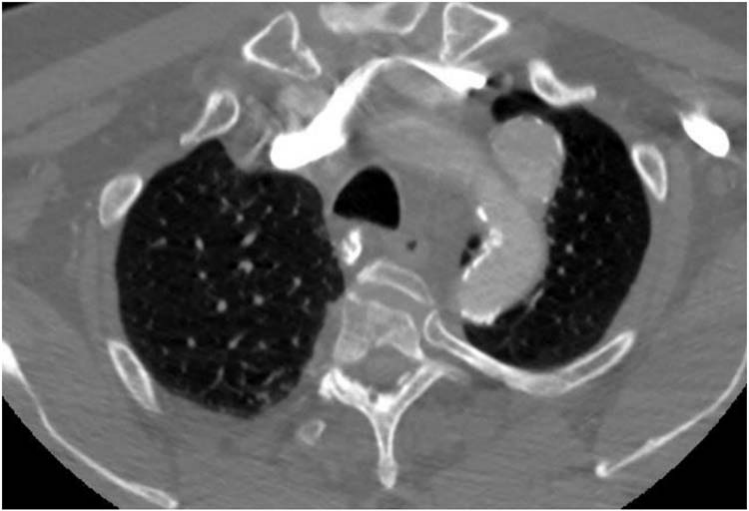
Preoperative CTA demonstrating LSA aneurysm; located at the origin from the aortic arch.

**Figure 2:**
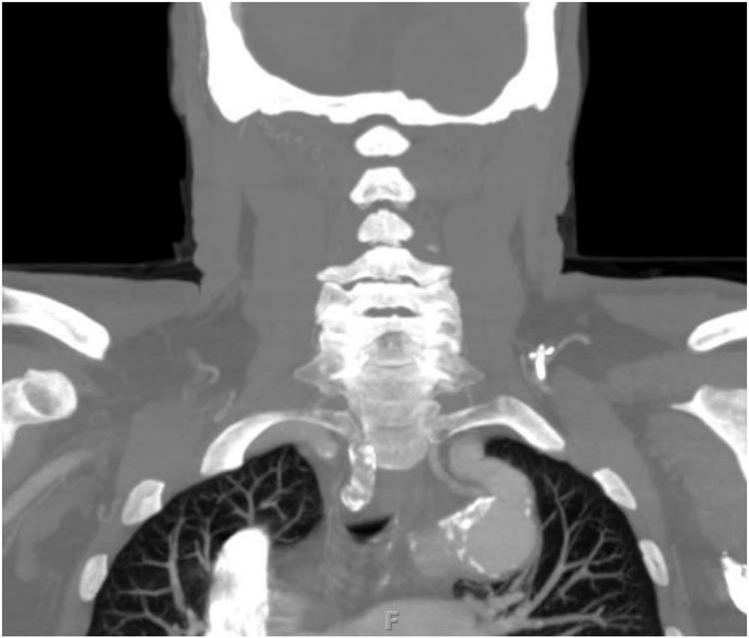
Preoperative CTA demonstrating aberrant left vertebral artery originating from the aortic arch, just proximal to the LSA.

The patient is a 70-year-old male with an incidental finding of a proximal LSA aneurysm on computed tomography (CT) angiography of the chest performed for shortness of breath. He had no dysphagia, neurologic or vascular occlusive symptoms. He had no history of trauma or known congenital anomaly. The aneurysm had a maximal diameter of 3.4 cm and was located at the origin of the artery (Fig. 1). An aberrant left vertebral artery and hypoplastic right vertebral artery were also identified. The left vertebral artery originated from the aortic arch proximal to the LSA takeoff (Fig. 2) and provided dominant posterior cerebral circulation.

**Figure 3:**
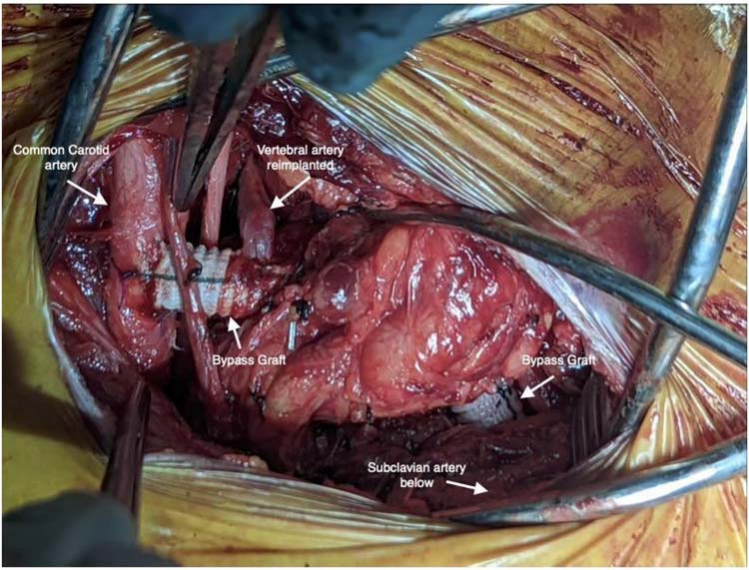
Intraoperative photo depicting left carotid to left subclavian bypass graft with left vertebral artery transposition into the graft.

**Figure 4:**
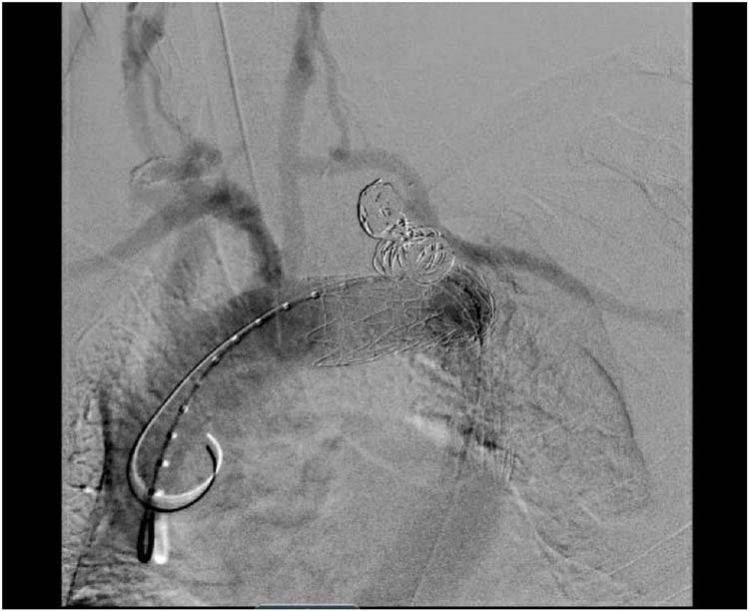
Aortic arch angiogram showing patency of the left carotid to subclavian artery bypass graft with vertebral artery implantation and showing coil embolization of the proximal left SAA.

Staged operative repair was utilized, where the first stage involved LSA revascularization. The patient underwent a left common carotid artery to LSA bypass utilizing a 7-mm Dacron graft. We performed left vertebral artery transposition onto the bypass graft in an end-to-side fashion (Fig. 3). Intraoperative doppler confirmed biphasic signals through the bypass graft and vertebral artery. The second stage was performed 3 days post-operatively, which included TEVAR with stent graft placement and coverage of LSA. After bilateral percutaneous common femoral artery access was obtained, arch aortogram was completed, which showed patency of the common carotid to LSA bypass and left vertebral artery. Embolization of the left SAA was performed using multiple Terumo coils™ (framing coil, azur cx coil, peripheral coil and helical hydrocoil). Next, a Gore C-Tag graft measuring 34 × 34 × 150 mm was deployed in Zone 2, just proximal to the take-off of the left common carotid artery. Repeat arch aortogram showed complete exclusion of the left SAA with a patent left common carotid artery, left carotid-subclavian bypass and left vertebral artery (Fig. 4). There was no further filling of the left SAA.

**Figure 5:**
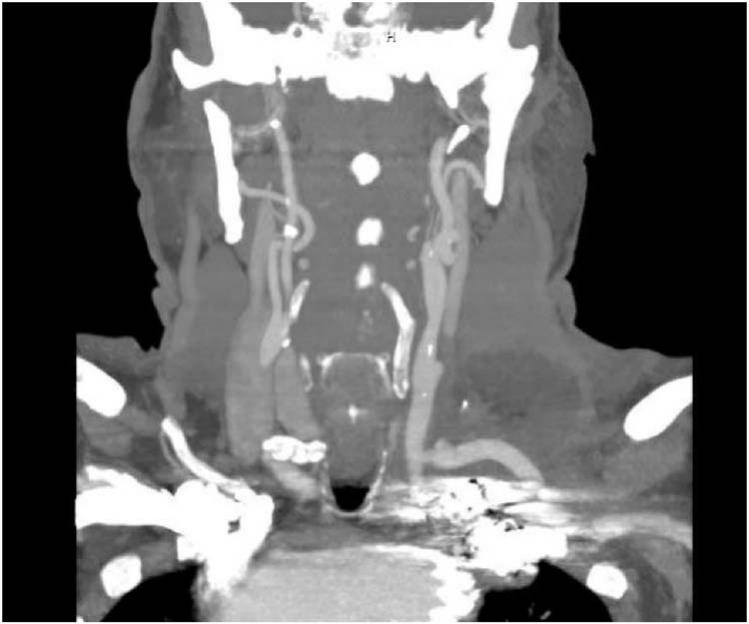
CT angiogram showing patency of the left carotid to subclavian artery bypass graft with vertebral artery implantation and showing coil embolization of the proximal left SAA.

The patient’s post-operative course was uneventful, and he was discharged home on post-operative day 6 from the initial operation and on post-operative day 3 from TEVAR. CT angiography done prior to discharge revealed successful exclusion of the left SAA without endoleak and a patent left common carotid to left subclavian bypass and transposed left vertebral artery (Fig. 5). The patient had repeat imaging 3 months post-procedure that again redemonstrated the above findings.

## DISCUSSION

SAAs are a rare entity that most commonly present with symptoms of local compression or are incidentally identified [3]. Local compression can result in brachial plexopathy, Horner’s syndrome, facial anhidrosis, hoarseness from recurrent laryngeal nerve compression, dysphagia or dyspnea due to involvement of the esophagus or trachea as well as venous obstruction [2]. The incidence of rupture is around 9% with an associated mortality rate of 19% [3]. Open repair via sternotomy or thoracotomy carries a high rate of complications and mortality [2]. Fortunately, the majority of SAA’s are amenable to endovascular approach to repair with an associated reduction in operative mortality [2, 3]. There are no guidelines for a size cutoff of SAA that should be repaired due to a paucity of data on the risk of rupture relative to the aneurysm size. Some suggest a 30-mm cutoff [4], while others recommend repair whenever feasible regardless of size [5].

Endovascular repair of SAA involves embolization of the aneurysm with concurrent TEVAR. The LSA is covered in up to 50% of TEVAR cases to achieve an adequate proximal landing zone and graft seal free of disease [6]. The LSA is the main arterial inflow to the left upper extremity, contributes to the posterior cerebral circulation through the vertebral artery and provides coronary circulation in patients with left internal mammary artery bypass graft. LSA coverage is often well tolerated because of the collateral circulation that is present. However, patients may have complications as a result of coverage; these include left upper extremity ischemia, posterior circulation stroke and spinal cord ischemia [7]. The decision to perform LSA revascularization can be divided into three distinctions: preoperative, intraoperative and post-operative. Preoperatively, the presence of a patent pedicled LIMA graft or left arm hemodialysis access, dominant left vertebral artery or origin from the aortic arch and spinal cord protection [7]. Intraoperative indications include evidence of left arm ischemia following device deployment, which is evident by loss of pulsatility in arterial monitoring [7]. Lastly, post-operative revascularization should be performed in the presence of left arm claudication or vertebrobasilar insufficiency [7]. The Society for Vascular Surgery practice guidelines recommend preoperative revascularization in the form of bypass or transposition in elective TEVAR patients where a proximal seal necessitates coverage of the LSA, or who have anatomy that compromises perfusion to critical organs [8]. Both of these recommendations are made despite very-low quality evidence.

In our case, the patient was also found to have an aberrant left vertebral artery originating off the aortic arch proximal to the subclavian artery. This is an uncommon anatomical variation that occurs in ~2–6% of the population [9, 10]. To our knowledge, there are only two other case reports describing carotid-subclavian bypass with vertebral artery transposition. These operations were not performed for repair of a SAA but rather for Type B aortic dissection and penetrating aortic ulcers with intramural hematoma [11, 12]. Blumberg *et al*. discusses a patient who was found to have a large intramural hematoma extending from the distal aortic arch to the descending aorta with two penetrating ulcers just distal to the takeoff of the LSA. They performed an end-to-side left vertebral artery to common carotid artery transposition, with left common carotid to LSA bypass using Gortex PTFE™ graft and with concomitant TEVAR. Chaney *et al*. present a patient found to have an extensive Type B aortic dissection originating from the proximal descending thoracic aorta just distal to the take-off of the LSA. They performed LSA to left common carotid transposition. This was followed by left vertebral to left subclavian transposition (carotid–subclavian–vertebral artery transposition) followed by TEVAR 2 weeks later.

## CONCLUSION

SAAs account for only a small percentage of aneurysms. The majority of these are amenable to endovascular repair. We present a unique SAA with aberrant left vertebral artery, in a patient with left side dominant posterior cerebral circulation, precluding safe coverage of the vertebral artery. The SAA was repaired in a staged hybrid approach; first with LSA revascularization and left vertebral transposition followed by a TEVAR.
